# Viral Features in a Twin Case of Severe Respiratory Syncytial Virus Infection

**DOI:** 10.1007/s12098-018-2831-9

**Published:** 2019-01-07

**Authors:** Yasuyo Kashiwagi, Masahiro Kimura, Tomoko Maeda, Soken Go, Hisashi Kawashima, Akihito Sawada, Tetsuo Nakayama

**Affiliations:** 1 Department of Pediatrics, Tokyo Medical University, 6-7-1 Nishishinjuku, Shinjuku-ku, Tokyo, 160-0023 Japan; 2 Kitasato Institute for Life Sciences, Laboratory of Viral Infection, Tokyo, Japan

*To the Editor:* Two male monochorionic diamniotic twins were born at a general hospital by cesarean section delivery at 37 wk and 5 d of gestation. The antenatal period of the twins was uneventful. The birth weights were 2424 g (first twin) and 2516 g (second twin), respectively. At 9 d, they were admitted to our pediatric ward due to the mother’s social factor.

They showed the upper respiratory symptoms such as cough and sneezing at day 21.

The results of respiratory syncytial virus (RSV) rapid assay with RSV antigens based on immunochromatography with nasal fluid (Check RSV; Alfresa, Japan) were positive. Their respiratory distress deteriorated and they required mechanical ventilation on day 25 (first twin) and day 22 (second twin). The duration of mechanical ventilation (first twin) was 8 d and the duration of mechanical ventilation (second twin) was 23 d due to severe respiratory distress. Their nasopharyngeal aspirate samples in the acute phase were examined using real-time RT-PCR [1]. Real-Time RT-PCR analysis detected high mounts of RSV type B, 1.7 × 10^5^ copies/viral RNA 1 μg (first twin) and 1.3 × 10^6^ copies/viral RNA 1 μg (second twin), respectively.

The levels of IL-8 were measured using a Bio-Plex suspension array (Bio-Rad Laboratories, Tokyo, Japan) in acute nasopharyngeal aspirate samples. IL-8 in the second twin (2421.59 pg/ml) was higher than that in the first twin (591.53 pg/ml).

RSV infection induces respiratory tract neutrophil response, IL-8, which is a major chemotactic factor for neutrophils and which is supposed to be a deleterious immune molecule in RSV infection [2].

In the second twin whose clinical course was more severe, RSV copy number and IL-8 in acute nasopharyngeal aspirate samples were high.

RNA copy number and IL-8 in the acute phase may have a role to assess the severity of RSV infection.
